# Chromatin Remodeling Enzyme Cluster Predicts Prognosis and Clinical Benefit of Therapeutic Strategy in Breast Cancer

**DOI:** 10.3390/ijms24065583

**Published:** 2023-03-15

**Authors:** Chia-Yu Kuo, Sin-Hua Moi, Ming-Feng Hou, Chi-Wen Luo, Mei-Ren Pan

**Affiliations:** 1 Division of Breast Oncology and Surgery, Department of Surgery, Kaohsiung Medical University Hospital, Kaohsiung 807, Taiwan; 2 Graduate Institute of Medicine, College of Medicine, Kaohsiung Medical University, Kaohsiung 807, Taiwan; 3 Graduate Institute of Clinical Medicine, Kaohsiung Medical University, Kaohsiung 807, Taiwan; 4 Research Center for Precision Environmental Medicine, Kaohsiung Medical University, Kaohsiung 807, Taiwan; 5 Department of Biomedical Science and Environmental Biology, College of Life Science, Kaohsiung Medical University, Kaohsiung 807, Taiwan; 6 Department of Cosmetic Science and Institute of Cosmetic Science, Chia Nan University of Pharmacy and Science, Tainan 717, Taiwan; 7 Center for Cancer Research, Kaohsiung Medical University Hospital, Kaohsiung 807, Taiwan; 8 Drug Development and Value Creation Research Center, Kaohsiung Medical University, Kaohsiung 807, Taiwan

**Keywords:** chromatin remodeling enzyme, epigenetic modification, chemotherapy, radiotherapy, hierarchical clustering

## Abstract

The treatment provided for breast cancer depends on the expression of hormone receptors, human epidermal growth factor receptor-2 (HER2), and cancer staging. Surgical intervention, along with chemotherapy or radiation therapy, is the mainstay of treatment. Currently, precision medicine has led to personalized treatment using reliable biomarkers for the heterogeneity of breast cancer. Recent studies have shown that epigenetic modifications contribute to tumorigenesis through alterations in the expression of tumor suppressor genes. Our aim was to investigate the role of epigenetic modifications in genes involved in breast cancer. A total of 486 patients from The Cancer Genome Atlas Pan-cancer BRCA project were enrolled in our study. Hierarchical agglomerative clustering analysis further divided the 31 candidate genes into 2 clusters according to the optimal number. Kaplan–Meier plots showed worse progression-free survival (PFS) in the high-risk group of gene cluster 1 (GC1). In addition, the high-risk group showed worse PFS in GC1 with lymph node invasion, which also presented a trend of better PFS when chemotherapy was combined with radiotherapy than when chemotherapy was administered alone. In conclusion, we developed a novel panel using hierarchical clustering that high-risk groups of GC1 may be promising predictive biomarkers in the clinical treatment of patients with breast cancer.

## 1. Introduction

Breast cancer is the most prevalent cancer globally, with 2.26 million new cases diagnosed in 2020 [1]. Breast cancer treatment includes surgery, chemotherapy, radiotherapy, and targeted therapy, which are comprehensively administered according to tumor size, lymph node status, and the expression of hormone receptors and human epidermal growth factor receptor-2 (HER2). For non-metastatic breast cancer, which at the time of diagnosis accounts for approximately 90% of patients, the treatment goals are tumor elimination and recurrence prevention [2].

Systemic treatment for non-metastatic breast cancer is based on molecular subtypes. For patients with hormone receptor-positive breast cancer, standard endocrine therapy is administered for 5–10 years, according to menopausal status [3]. HER2-targeted antibodies are effective in preventing recurrence and increasing overall survival in patients with HER2-positive breast cancer [4]. Recently, several emerging antibody-drug conjugates have been approved by the U.S. Food and Drug Administration (FDA) and applied to breast cancer with HER2-overexpression. It has the advantage of delivering selective drugs to specific antigen-presenting cells, thereby minimizing toxicity to normal cells [5]. For patients who are lymph node positive, HER2-positive, or have triple-negative breast cancer (TNBC) and a tumor size of >1 cm, adjuvant anthracycline with cyclophosphamide and taxane-based chemotherapy is the backbone of treatment [6]. The neoadjuvant chemotherapy setting is applied to some patients with locally advanced breast cancer to facilitate breast conservation during surgery and can provide important prognostic information about the response to treatment, particularly in HER2-positive breast cancer and TNBC [7].

Clinically, prognostic factors can predict the population of at-risk patients, while predictive factors can identify a certain group that benefits from treatment. The strongest prognostic factors for breast cancer include tumor size, axillary lymph node involvement, histological grade, hormone receptor status, and age at diagnosis. The proportion of distant metastatic disease in breast carcinoma in situ was <1% at 10 years of age. The 5-year overall survival rates of stage I hormone receptor-positive breast cancer, HER2-positive breast cancer, and TNBC are >99%, >94%, and >85%, respectively [8]. As predictive factors for breast cancer, estrogen receptor (ER) modulators can predict the likelihood of response to hormone therapy, and HER2/neu can predict the value of monoclonal antibodies, such as trastuzumab, pertuzumab, and neratinib [9,10]. However, regarding adjuvant chemotherapy and radiotherapy, there is still a lack of powerful biological hallmarks to discern whether treatment will be beneficial.

Currently, oncologists use molecular genomic profiling tests to establish precision medicine that can help personalize treatment, avoid over- or under-treatment, and maximize the outcomes for all subtypes of breast cancers. The following are several commercially available modalities of genomic assays that help clinicians decide on systemic therapy for early invasive breast cancer unrelated to the National Comprehensive Cancer Network, including Oncotype DX [11], MammaPrint [12], Prosigna [13], EndoPredict [14], and breast cancer index (BCI) [15]. Multigene assays provide useful information, especially for patients with breast cancer who are equivocal for adjuvant therapy plans based on traditional immunohistochemical staining markers and clinical features. Notably, only a few genes are shared between the prognostic signatures, most of which differ. These genes do not include those involved in epigenetic modifications [16]. In fact, there is discordance between these molecular prognostic assays; the preliminary phase III OPTIMA trial found that approximately 60% of patients were categorized into different risk groups [17]. Moreover, ideal prognostic and predictive parameters for HER2-positive or TNBC are still lacking. Although distant recurrence can be calculated using basic clinical data, there was approximately a 20% risk of distant recurrence in patients in the low-risk group who did not receive adjuvant chemotherapy [18]. Thus, developing a promising prognostic or predictive assay not only determines the benefits of chemotherapy and radiation therapy but also provides information on effective new drugs.

To date, researchers have investigated the relationship between epigenetic alterations and tumorigenesis. Recent studies have shown that epigenetic modifications are important in human breast cancer [19,20]. This process contributes to carcinogenesis by activating oncogenes and silencing tumor suppressor genes, thereby driving tumor growth and invasion [20,21]. As epigenetic modifications have been established, epigenetic-associated biomarkers may potentially serve as predictive or prognostic targets for the treatment of breast cancer [22]. HDAC (Histone Deacetylases) and DNMT (DNA Methyltransferases) are epigenetic modifiers that regulate gene expression by altering chromatin structure [23]. PRDM (PR/SET Domain) and PRMT (Protein Arginine Methyltransferase) families are transcriptional regulators that control gene expression by modifying histones and other proteins [24]. Aberrant expression of these genes has been linked to the development and progression of breast cancer [19]. Elevated levels of HDAC and DNMT have been associated with poor prognosis and resistance to chemotherapy and radiotherapy in breast cancer patients [25]. On the other hand, decreased expression of PRDM and PRMT genes has been linked to poor prognosis and resistance to therapy [24].

It is thought that changes in the expression levels of these genes alter the expression of critical genes involved in cell cycle regulation, DNA damage repair, and apoptosis. These alterations may affect the sensitivity of cancer cells to chemotherapy and radiotherapy and contribute to the development of resistance. However, the mechanism behind the association of these genes with breast cancer patient outcomes and response to therapy is complex and not fully understood. In the past decade, the direction of cancer treatment is from a “one-size-fits-all” approach turn into focusing on the precision medicine based on genomic variants [26]. Therefore, we focused on common, well-known 5 proteins of DNMT family, 3 proteins of HDAC family, 15 proteins of PRDM family, and 8 proteins of PRMT family to discover an effective prognostic or predictive factor focusing on epigenetic processing of breast cancer in chemotherapy and radiotherapy settings using hierarchical agglomerative clustering analysis.

## 2. Results

### 2.1. Baseline Characteristics

A total of 486 patients were included in this study. Table 1 shows the baseline characteristics of the study groups. The mean age of the patients was 55.9 years old and 68% were aged >50 years. Among all the patients, 106 (22%) had TNBC and 380 (78%) had non-TNBC. A total of 261 patients (54%) presented with lymph node invasion. According to the eighth edition of the AJCC TNM system, 300 patients (62%) had pathological stage II disease, 87 (18%) had stage I disease, and 99 (20%) had stage III disease. Of the patients, 305 (63%), 386 (79%), and 253 (52%) received radiotherapy, chemotherapy, and a combination of the two, respectively. In total, 29 (6.0%) of the patients died, and disease progression was observed in 45 (9.3%) patients.

### 2.2. Hierarchical Clustering Analysis

**Figure 1:**
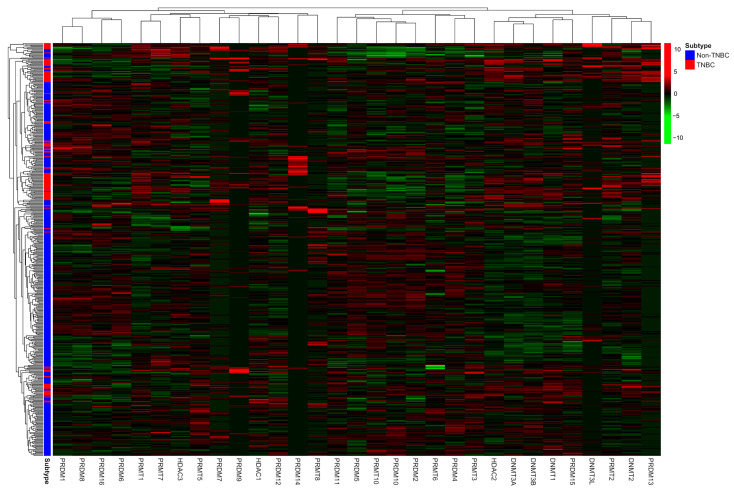
Heatmap of normalized mRNA expression of 31 candidate genes according to molecular subtype of study population. Red color indicates higher expression level, while green color indicates lower expression level of mRNA expression.

**Figure 2:**
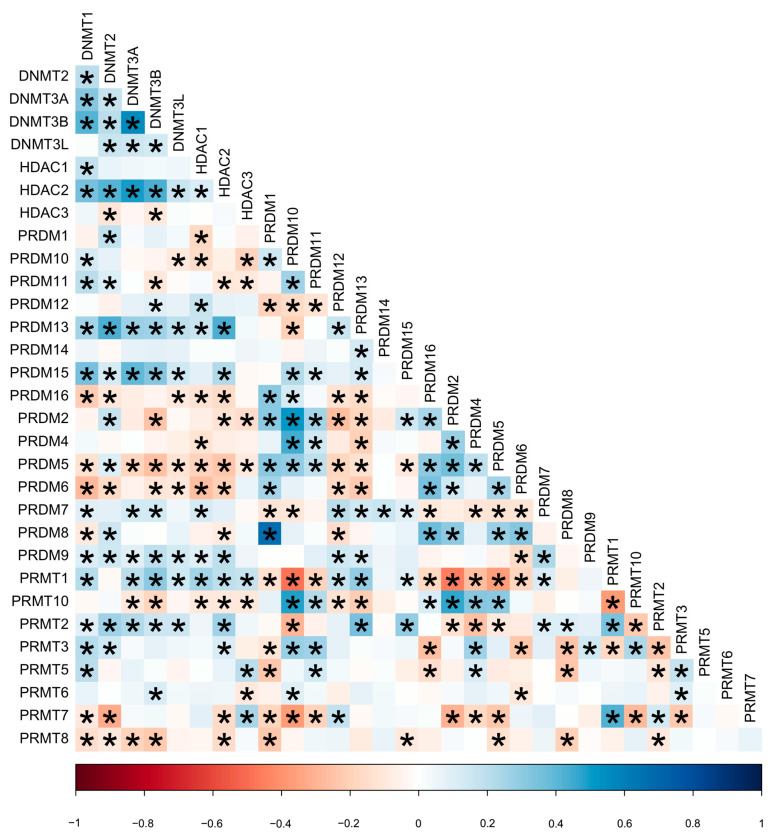
Heatmap of correlation between 31 candidate genes in all study population. * *p*-value less than 0.05 estimated using Pearson correlation test. Red color indicates negative correlation (−1); blue color indicates positive correlation (+1); and the color intensity represents the strength of correlation.

**Figure 3:**
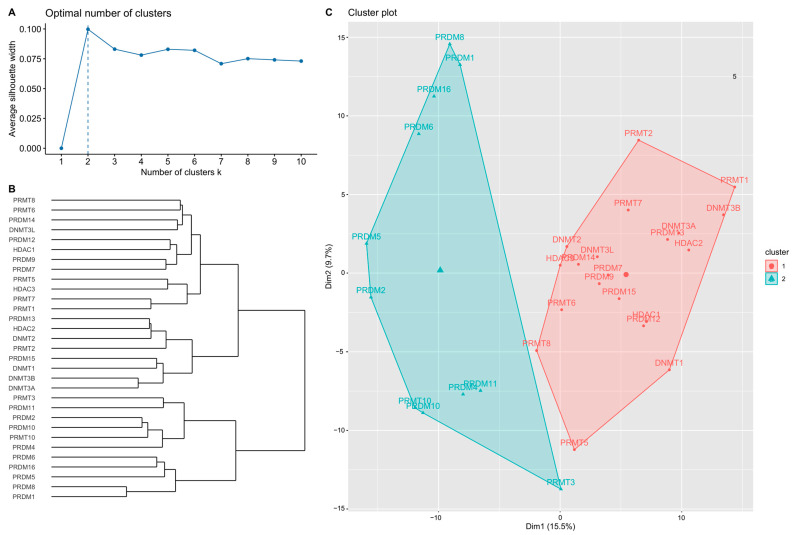
Hierarchical clustering results of the target genes. (**A**) Optimal number of clusters estimated according to silhouette index. (**B**) Dendrogram of hierarchical clustering results of 31 target genes. (**C**) Cluster plot of target genes using the optimal number of clusters.

The mRNA expression of the 31 candidate genes in the study population is shown in Table 2 and normalized using a heatmap (Figure 1). The median and range of each gene were calculated and compared with those of normal tissue. High and low expression of each gene are represented with a red-to-green color bar among the total 486 patients in the TNBC and non-TNBC groups. Figure 2 shows a heatmap of the correlations among the 31 candidate genes in all study populations. A high-intensity positive correlation (blue) was observed between *DNMT3B/DNMT3A, HDAC2/DNMT3A, PRDM2/PRDM10, PRDM4/PRDM10, PRDM8/PRDM1, PRMT10/PRDM10, PRMT10/PRDM2*, and *PRMT7/PRMT1*. A strong negative correlation (red) was observed between *PRMT1/PRDM10, PRMT1/PRDM2, PRMT1/PRDM5, PRMT7/PRDM10,* and *PRMT10/PRMT1*. Figure 3 shows the results of the hierarchical clustering analysis. According to the silhouette index, the optimal number of clusters was two (Figure 3A). A dendrogram of the target genes is shown in Figure 3B. All 31 target genes were assigned to 2 gene clusters according to the optimal number of clusters, and are illustrated in a cluster plot in Figure 3C.

### 2.3. Two Clusters According to the Optimal Number in Analysis

According to mRNA expression levels, the target genes were distributed into 2 groups, named gene clusters 1 and 2 (GC1/2). GC1 contained 20 genes, including *DNMT1, DNMT2, DNMT3A, DNMT3B, DNMT3L, HDAC1, HDAC2, HDAC3, PRDM7, PRDM9, PRDM12, PRDM13, PRDM14, PRDM15, PRMT1, PRMT2, PRMT5, PRMT6, PRMT7*, and *PRMT8*. GC2 contained 11 genes: *PRDM1, PRDM2, PRDM4, PRDM5, PRDM6, PRDM8, PRDM10, PRDM11, PRDM16, PRMT3*, and *PRMT10*. Risk estimation subgroups according to overall patient characteristics, lymph node invasion status, and treatment were further analyzed.

### 2.4. Higher mRNA Expression of Target Genes in High-Risk Groups

The high-risk subgroups in GC1 and GC2 revealed higher mRNA expression of almost all target genes, as shown in Table 3 and Table S1, with significant differences (*p* < 0.05), except for *HDAC2, PRDM13, PRMT2, PRMT5, PRMT6*, and *PRDM11*. In GC1, *DNMT1*, *DNMT2, DNMT3A, DNMT3B, HDAC1, HDAC2, HDAC3, PRDM12, PRDM13, PRDM15, PRMT1, PRMT2*, and *PRMT5* showed positive values in the high-risk subgroup, whereas *DNMT3L, PRDM7, PRDM9, PRDM14, PRMT7*, and *PRMT8* showed negative median values. In GC2, *PRDM1, PRDM4, PRDM6*, and *PRMT3* had positive median values in the high-risk subgroup, whereas *PRDM2, PRDM5, PRDM8, PRDM10, PRDM11, PRDM16*, and *PRMT10* had negative median values.

### 2.5. High- and Low-Risk Groups Clinical Manifestation

The distributions of the baseline characteristics according to hierarchical clusters are summarized in Table S2. The distribution of TNBC was significantly different between the low- and high-risk groups for both GCs (*p* < 0.001). There were no significant differences in lymph node invasion, stage, or radiotherapy between the low- and high-risk groups within the two GCs. More than 90% of high-risk patients in GC1 received chemotherapy, with a significant difference (*p* < 0.001). Progressive disease was more frequent in the high-risk group of GC1 compared with the low-risk group (16 [16.5%] vs. 29 [7.5%], *p* = 0.006).

### 2.6. Progression-Free Survival Is Lower in the High-Risk Group of GC1

**Figure 4:**
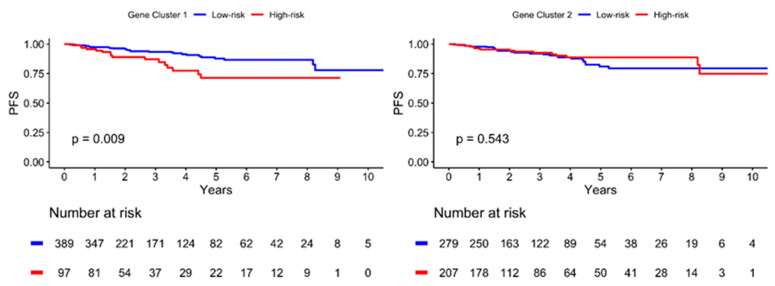
Kaplan–Meier plot for progression-free survival (PFS) according to risk estimation subgroup of two gene cluster determined using hierarchical clustering analysis. Red solid line indicates high-risk subgroup, and blue solid line indicates low-risk group. *p*-value is estimated using log-rank test.

**Figure 5:**
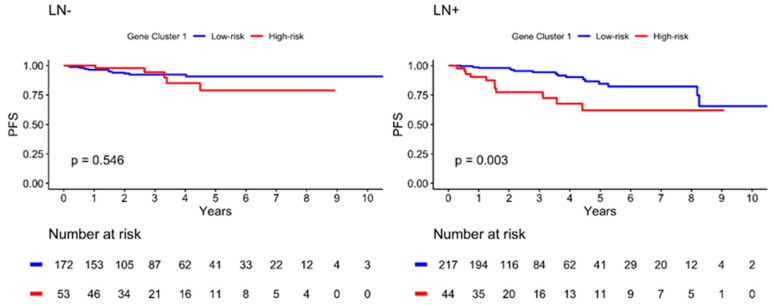
Kaplan–Meier plot for PFS according to risk estimation subgroup of gene cluster 1 in different lymph node (LN) invasion status. Red solid line indicates high-risk subgroup, and blue solid line indicates low-risk group. *p*-value is estimated using log-rank test.

**Figure 6:**
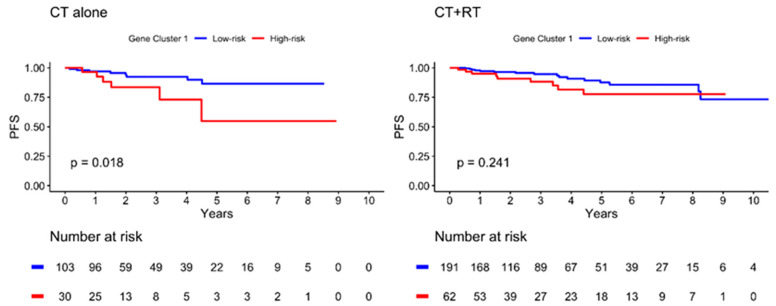
Kaplan–Meier plot for PFS according to risk estimation subgroup of gene cluster 1 in different treatment subgroup. Red solid line indicates high-risk subgroup, and blue solid line indicates low-risk group. *p*-value is estimated using log-rank test.

The Kaplan–Meier plot illustrating the 10-year PFS is shown in Figure 4. The patients were further divided into low- and high-risk subgroups in both gene clusters according to the risk estimation analysis. The results showed significant differences between the low- and high-risk groups in GC1 (*p* = 0.009), with a lower PFS in the high-risk group. However, no significant differences were observed in GC2 (*p* = 0.543). In addition, the high-risk GC1 group with lymph node invasion showed worse PFS than the low-risk group (*p* = 0.003), as illustrated in Figure 5. The estimated risk subgroup of GC1 revealed a worse PFS at high risk of chemotherapy alone (*p* = 0.018). Interestingly, there was a trend toward better PFS in the high-risk subgroup of GC1 in the chemotherapy combined with radiotherapy setting, but the difference was not significant (*p* = 0.241) (Figure 6). Additionally, chemotherapy with radiotherapy seemed to have a better PFS in the high-risk GC1 group than in the low-risk group, although the difference was not significant (*p* = 0.214) (Figure S1).

### 2.7. mRNA Expression and Progression-Free Survival Analysis of GC1 High-Risk Group Target Genes According to TNBC and Non-TNBC Subgroups

The boxplot of the 20 target genes of the high-risk group in GC1 demonstrated significant differences in mRNA expression in the TNBC and non-TNBC groups, except for *HDAC3*, *PRDM14*, *PRDM9*, and *PRMT6* (Figure S2). PFS was inferior in the high-risk group of GC1 in the TNBC subgroup analysis, although the difference was not significant (*p* = 0.061) (Figure S3). Regarding lymph node status, there was a trend for worse PFS in the high-risk group for GC1 in TNBC with lymph node invasion (Figure S4). Combined chemotherapy with radiotherapy seemed to result in better PFS in the TNBC subgroup with high-risk GC1 genes, as seen in the lymph node invasion analysis (Figures S5–S8).

## 3. Discussion

Breast cancer is a complex and multifactorial disease and the most common cancer in women worldwide [27]. Currently, its diagnosis relies on analysis of clinical manifestations, imaging scans, and immunohistopathological examinations. Among all breast cancer subtypes, TNBC has a poor prognosis and high metastatic ability owing to its heterogeneity [28]. Conventional systemic chemotherapy remains the mainstay of treatment. Radiotherapy is reserved for breast conservative surgery or post-mastectomy high-risk patients as a local, regional control. Recently, some targeted therapies and the immune checkpoint inhibitors were developed for advanced TNBC treatment [29,30]. However, not all TNBC patients respond to these treatments [31]. Therefore, it still needs to find more precise treatments for individuals.

In the present study, a serial comprehensive analysis was conducted based on hierarchical clustering results. Thirty-one genes were divided into two clusters, thereby fulfilling the optimal number. We found that the high-risk group in GC1 had a higher proportion of TNBCs than the low-risk group. Moreover, patients in the high-risk subgroup of GC1 displayed a significantly lower PFS than those in the low-risk subgroup (*p* = 0.009). Another interesting finding was that combining chemotherapy with radiotherapy might result in a better survival rate in the high-risk subgroup of GC1. Hence, the panel of high-risk gene expression in GC1 may play a role as a promising predictive index and may also be effective in predicting treatment response to chemotherapy combined with radiotherapy in patients with TNBC. The risk estimation effects of GC1 maintained a consistent threshold in the TNBC and TNBC-LN-positive subgroups. Although no significant differences were found, the high-risk subgroup was more likely to achieve a poor PFS than the low-risk subgroup. The marginal effects of statistical significance may be because of the restricted sample size.

Epigenetic processes play important roles in carcinogenesis. The most well-known and comprehensive study of epigenetic mechanisms involves DNA methylation. Alterations in DNA methylation can lead to the development of cancer. Previous study indicated that *DNMT1*, *DNMT3A*, and *DNMT3B* are upregulated in breast tumor tissues compared with normal breast tissues [32]. In addition, higher expression of *DNMT1* and *DNMT3A* was found in TNBC than in other breast cancer subtypes. Consistent with these findings, we found that the target genes were associated with epigenetic processing.

One of the key mechanisms involved in the regulation of histone modification is histone acetylation and deacetylation by *HAT* and *HDAC*, respectively. Several studies have identified deregulation of HDAC, particularly in breast cancer. Previous study found that *HDAC1* and *HDAC3* were upregulated in *E2F1* and *E2F4*, which are responsible for the downregulation of *ARH1*, a maternally imprinted tumor suppressor gene [33]. Another comparative study demonstrated that global hypoacetylation of H4 was observed in the progression from normal epithelium to ductal carcinoma in situ to invasive ductal carcinoma [34]. However, the abovementioned studies only focused on analyzing genes or proteins from one of the chromatin-modifying enzymes individually, which were different to this study.

Protein arginine methyltransferase (*PRMT*) is a histone methyltransferase (HMT) that methylates glycine- and arginine-rich motifs (GAR motifs). *PRMTs* are classified into three groups according to their differences in catalytic activity: type I (*PRMT1, 2, 3, 6*, and *8*, *CARM1* [*PRMT4*]), type II (*PRMT5* and *9*), and type III (*PRMT7*). Next, we focused on discussing the relationship among *PRMT2*, *PRMT8, PRMT7*, and previous findings. The mRNA expression analysis of *PRMT2* showed a negative median range in patients with breast cancer, and the high-risk group of GC1 reflected disputable consequences in this study. Previous studies also indicated that the role of *PRMT2* in breast cancer progression remains controversial. A study suggested that spliced variants of *PRMT2* are capable of binding to ERα, which further promotes EHMTRα-mediated EMT-related genes E-cadherin and Snail [35]. However, other studies indicated that the loss of PRMT2 may be essential for increasing the invasiveness of breast cancer [36,37]. Therefore, it is necessary to further study the function of *PRMT2* in breast cancer. PRMT8 is an enzyme whose exact mechanism of action in breast cancer is poorly understood. Previous study indicated that PRMT8 was associated with a poor prognosis via z-score analysis, whereas the Kaplan-Meier plot showed a protective trend for breast cancer [38]. Another study observed that high *PRMT8* expression correlated with worse survival in gastric cancer; however, increased survival was observed in patients with breast and ovarian cancer. These contrasting results may be attributed to variant-specific expression of PRMT8, including the novel *PRMT8* variant 2 [39]. This may explain why *PRMT8* in GC1 was negatively expressed in both the high- and low-risk subgroups. E-cadherin, an essential biomarker of EMT, is downregulated by *PRMT7* at its promoter, with elevated H4R3me3 and decreased H3K4me3, H3, and H4Ac. *PRMT7* interferes with E-cadherin expression through the PRMT7-YY1-HDAC3 complex, thus inducing cell migration and invasion in the MDA-MB-231 TNBC cell line [40]. However, the results of our study showed higher expression of *PRMT7* in the low-risk subgroup of GC1, which may indicate an insidious pathway or variations in *PRMT7* enzyme activity.

To date, the *PRDM* families were found to contain 19 proteins that presented with a PR domain in the N-terminal region and variable numbers of zinc finger repeats [24]. Hierarchical clustering analysis selected 6 PRDMs (*PRDM7, 9, 12, 13, 14, 15*) in GC1, and half of them showed negative expression (*PRDM7, 9, 14*), whereas the rest showed positive expression (*PRDM12*, *13*, *15*) in the high-risk group. However, studies of PRDM7 and carcinogenesis are limited. Sorrentino et al. observed that PRDM7 was overexpressed in hepatocarcinoma specimens through pan-cancer reanalysis of TCGA datasets. PRDM9 has been reported to interfere with homologous recombination by binding DNA using its zinc fingers, and H3K4/36me3 occurs in surrounding nucleosomes [41]. It involves overlapping mechanisms of DSB during meiosis and mitosis [42,43]. Mutations in *PRDM9* have yet to be correlated with some solid tumors, such as head and neck squamous cell carcinoma and bladder cancer [44,45]. Previous studies suggested that *PRDM12* may act as a tumor suppressor in patients with CML [46]. However, several studies indicated that *PRDM12* is upregulated in prostate and colon cancers compared to normal tissues [47]. *PRDM13* acts as an anti-proliferation regulator by upregulating *INCA1*, a CDK inhibitor, and *ADAMTS12*, thereby inhibiting cell invasion. Overexpression of *PRDM13* in U87 glioma cells significantly inhibits cell migration and invasion by interacting with deleted liver cancer 1 (DLC1) and ARHGAP30 (Rho GTPase-activating protein 30) genes [48]. However, pan-cancer reanalysis has shown high *PRDM13* overexpression in many cancers. These results are consistent with our study, in which *PRDM13* expression was higher in the high-risk subgroup of GC1. *PRDM15* could be a key regulator in diffuse large B-cell lymphoma (DLBCL), which sustained the activity of the PI3K/AKT/mTOR pathway and glycolysis [49]. In addition, *PRDM15* could also stabilize the naïve pluripotency of embryonic development by modulating the transcription of upstream regulators of WNT and MAPK-ERK signaling [50]. Previous studies found that there is elevated expression of *PRDM14* in breast cancer, whereas low or no expression was found in non-tumorous cells. Moreover, its amplification has been correlated with more aggressive cases of breast cancer, including high-grade and distant metastases. Knockdown of *PRDM14* suppressed cell growth by inducing apoptosis and increasing sensitivity to chemotherapy agents [51,52,53]. However, *PRDM14* mRNA expression was negative for both high- and low-risk GC1 in our study. Evidence has shown that significant *PRDM14* methylation occurs in high-grade non-muscle invasive bladder, colon, and lung cancers. *PRDM14*′s role as a tumor suppressor—and whether its variant isoform exists—remains unknown [54,55,56].

Based on the provided information, our study found that the expression of a combination of genes, including *DNMT1, DNMT2, DNMT3A, DNMT3B, DNMT3L, HDAC1, HDAC2, HDAC3, PRDM7, PRDM9, PRDM12, PRDM13, PRDM14, PRDM15, PRMT1, PRMT2, PRMT5, PRMT6, PRMT7*, and *PRMT8*, could be associated with an increased risk of poor PFS in patients with lymph node invasion. Additionally, the study suggested that the combination of chemotherapy and radiotherapy may be more effective in improving PFS compared to chemotherapy alone. We also tried to determine crosstalk between pathways with four gene families, including DNMT, HDAC, PRMT, and PRDM, which were illustrated using Ingenuity pathway analysis (IPA) systems (Figure S9). The possible mechanisms underlying these findings are not clear from the information provided, but some of the genes identified in the study are known to be involved in epigenetic regulation and protein modification. It is possible that the combination of these genes could be involved in the regulation of key pathways involved in cancer progression, such as cell proliferation, apoptosis, and DNA repair. Further studies would be needed to confirm these findings and to investigate the underlying mechanisms in more detail.

The limitations of our study are the relatively small sample size and the lack of clinical practice. Nonetheless, the study gives us an indication that epigenetic modifications may play a crucial role in breast cancer tumorigenicity and could be a therapeutic target in the future.

## 4. Material and Methods

### 4.1. Data Source

All data were downloaded from the TCGA Pan-Cancer BRCA project via the cBioPortal platform [57,58]. This study included patients with non-metastatic breast cancer diagnosed as invasive ductal carcinoma by histological confirmation. Baseline characteristics included age at diagnosis, subtype, stage of tumor size, lymph node invasion status, pathological stage, radiotherapy, and chemotherapy. Progression-free survival (PFS) was considered as the primary endpoint of the study population. All patients were tracked from the date of initial diagnosis to the date of disease progression, metastasis, or the end of the study.

### 4.2. Messenger RNA (mRNA) Expression

The differential expression of candidate genes was estimated using raw digital gene expression counts, which were normalized using the variation of the reads/Kb/Million (RPKM). mRNA expression indicates the RPKM-normalized log2-transformed differential expression profiles (comparing the expression z-scores of tumor samples and adjacent normal samples in the study cohort) for the candidate genes. The mRNA expression of the candidate genes in all patients was visualized using a heatmap and the correlation between candidate genes were illustrated using a heatmap and tested using Pearson’s correlation test.

### 4.3. Hierarchical Clustering

The mRNA expression levels of candidate genes were normalized into a range of 0 to 1. The average silhouette width in *k* clusters was calculated to determine the optimal number of clusters for the candidate genes. Hierarchical clustering was conducted to generate dendrograms for candidate genes according to the gene similarity in mRNA expression. The greatest silhouette width was considered the optimal number of clusters to ensure the greatest dissimilarity between the identified *n* gene clusters. The cluster plot of candidate genes generated according to the assigned *n* gene clusters showed the similarity of the clustered genes in a two-dimensional distribution. Next, mRNA expression in the *n* gene cluster was used to dichotomize the study population into two risk estimation subgroups according to PFS status. The cutoff criteria for the two risk estimation subgroups were determined based on the distance metrics computed using a hierarchical algorithm based on the similarity of correspond mRNA expression. The computed distances were then used to establish cutoff values that separated the study population into two risk estimation subgroups, the subgroup with a higher proportion of disease progression was classified as the high-risk group, while the subgroup with a lower proportion was classified as the low-risk group.

### 4.4. Statistical Analysis

The baseline characteristics of all patients and the risk estimation subgroup determined by each gene cluster were summarized as frequency and percentage, and age at diagnosis was summarized by mean and standard deviation. The difference in baseline characteristic distribution between the risk estimation subgroups was estimated using an independent two-sampled t-test or chi-squared test. The mRNA expression of all candidate genes in all patients and the risk estimation subgroup were summarized using medians and ranges. The difference in mRNA expression between the risk-estimation subgroups was estimated using the Wilcoxon rank-sum test. The PFS between risk estimation subgroups in all patients, different lymph node invasion status, and treatment groups was estimated using the Kaplan–Meier method, and the survival difference between subgroups was tested using the log-rank test. All *p*-values were two-sided, and statistical significance was set at *p* < 0.05. All analyses were performed using the R 4.0.5 software (R Core Team, Vienna, Austria, 2021).

## 5. Conclusions

In this study, we aimed to identify an optimal predictive biomarker for TNBC. First, we searched the TCGA database to select 486 patients, and 31 candidate genes were identified. The hierarchical clustering analysis represented two clusters according to the optimal number. GC1 was significantly associated with patients with breast cancer. Second, Kaplan–Meier analysis showed shorter PFS in the high-risk group of GC1 and lymph node invasion subgroups. The trend of improved PFS in the chemotherapy combined with radiotherapy group suggests that the high risk in GC1 genes may indicate better prognosis under treatment. Herein, we propose that the high-risk group genes in GC1 may be promising predictive biomarkers and predict clinical benefits from chemotherapy combined with radiotherapy.

## Figures and Tables

**Table 1:** Baseline characteristics of study population (*n* = 486).

Characteristics	Overall
Diagnosis age (years), mean ± SD	55.9 ± 12.4
Age group ≥ 50 years	329 (68%)
Age group < 50 years	157 (32%)
Subtypes	
Triple negative breast cancer (TNBC)	106 (22%)
Luminal A	224 (46.1%)
Luminal B	116 (23.9%)
HER2 type	40 (8.2%)
Pathological stage	
Stage I	87 (18%)
Stage II	300 (62%)
Stage III	99 (20%)
IIIA	70 (14.4%)
IIIB	6 (1.2%)
IIIC	23 (4.7%)
Stage IV	0 (0%)
Tumor size	
T1 (<2 cm)	144 (29.6%)
T2 (2 cm–5 cm)	303 (62.3%)
T3 (>5 cm)	32 (6.6%)
T4 (direct to chest wall or skin)	7 (1.4%)
Lymph node status	
Non-lymph node invasion (LN0)	225 (46%)
Lymph node invasion (LN+)	261 (54%)
N1 (1–3)	178 (36.6%)
N2 (4–9)	59 (12.1%)
N3 (≥10)	24 (4.9%)
Treatment	
Radiotherapy (RT)	305 (63%)
Chemotherapy (CT)	386 (79%)
Treatment subgroup	
RT alone	52 (10.7%)
CT alone	133 (27.4%)
Chemotherapy + Radiotherapy	253 (52%)
Survival outcome	
Died	29 (6%)
Disease progressed	45 (9.3%)

SD, standard deviation.

**Table 2:** mRNA expression of 31 candidate genes among study population.

Genes	mRNA Expression	Genes	mRNA Expression
*DNMT1*	1.75 (−1.75, 6.11)	*PRDM2*	−1.53 (−8.84, 2.85)
*DNMT2*	−2.06 (−8.62, 5.47)	*PRDM4*	0.53 (−7.29, 6.63)
*DNMT3A*	1.96 (−1.48, 6.86)	*PRDM5*	−2.83 (−11.00, 3.04)
*DNMT3B*	2.19 (−2.45, 7.70)	*PRDM6*	−0.13 (−4.58, 6.60)
*DNMT3L*	−3.12 (−3.12, 31.38)	*PRDM7*	-1.86 (-1.86, 6.63)
*HDAC1*	0.96 (−5.19, 4.74)	*PRDM8*	−2.58 (−9.36, 3.47)
*HDAC2*	1.38 (−4.16, 10.61)	*PRDM9*	−14.32 (−14.32, 56.65)
*HDAC3*	0.17 (−6.33, 5.73)	*PRMT1*	0.97 (−2.07, 4.77)
*PRDM1*	0.34 (−4.65, 4.93)	*PRMT10*	−1.51 (−8.33, 2.63)
*PRDM10*	−0.38 (−4.63, 3.37)	*PRMT2*	−1.14 (−4.80, 7.08)
*PRDM11*	−1.70 (−6.01, 2.43)	*PRMT3*	0.80 (−5.64, 7.34)
*PRDM12*	1.11 (−2.13, 7.80)	*PRMT5*	0.60 (−5.05, 8.60)
*PRDM13*	−2.24 (−2.24, 21.56)	*PRMT6*	0.66 (−6.83, 4.13)
*PRDM14*	−5.16 (−5.16, 10.82)	*PRMT7*	−0.001 (−3.79, 6.10)
*PRDM15*	0.50 (−3.15, 4.44)	*PRMT8*	−0.76 (−1.64, 14.55)
*PRDM16*	−2.80 (−6.56, 2.62)		

All mRNA expressions were summarized using median and range.

**Table 3:** mRNA expression of target genes according to risk estimation subgroup determined using GC1.

Genes	Gene Cluster 1		*p*
	Low-Risk (*n* = 389)	High-Risk (*n* = 97)	
Gene cluster 1 included			
*DNMT1*	1.59 (−1.75, 6.11)	2.39 (−0.68, 5.77)	<0.001
*DNMT2*	−2.37 (−8.62, 3.27)	0.01 (−7.78, 5.47)	<0.001
*DNMT3A*	1.74 (−1.48, 5.55)	3.15 (−0.68, 6.86)	<0.001
*DNMT3B*	1.71 (−2.45, 7.31)	3.64 (0.26, 7.70)	<0.001
*DNMT3L*	−3.12 (−3.12, 10.90)	−3.12 (−3.12, 31.38)	<0.001
*HDAC1*	0.89 (−5.19, 4.74)	1.22 (−0.81, 3.89)	<0.001
*HDAC2*	0.77 (−4.16, 8.34)	4.61 (−1.65, 10.61)	0.076
*HDAC3*	0.11 (−6.33, 5.73)	0.55 (−3.36, 4.82)	<0.001
*PRDM7*	−1.86 (−1.86, 6.63)	−1.86 (−1.86, 5.33)	<0.001
*PRDM9*	−14.32 (−14.32, 7.42)	−14.32 (−14.32, 56.65)	<0.001
*PRDM12*	0.94 (−2.13, 7.80)	1.90 (−2.13, 6.70)	<0.001
*PRDM13*	−2.24 (−2.24, 13.99)	3.12 (−2.24, 21.56)	0.373
*PRDM14*	−5.16 (−5.16, 10.82)	−5.16 (−5.16, 2.22)	<0.001
*PRDM15*	0.35 (−3.15, 3.16)	1.35 (−1.35, 4.44)	<0.001
*PRMT1*	0.72 (−2.07, 4.47)	1.81 (−1.95, 4.77)	<0.001
*PRMT2*	−1.30 (−4.80, 3.34)	0.25 (−3.32, 7.08)	0.077
*PRMT5*	0.67 (−5.05, 5.48)	0.33 (−3.67, 8.60)	0.229
*PRMT6*	0.66 (−6.83, 4.13)	0.63 (−3.88, 3.37)	0.308
*PRMT7*	0.002 (−3.75, 6.10)	−0.14 (−3.79, 3.80)	0.011
*PRMT8*	−0.68 (−1.64, 14.55)	−0.92 (−1.64, 10.46)	<0.001

All mRNA expressions were summarized using median and range. *p*-value is estimated using Wilcoxon rank-sum test.

## Data Availability

TCGA data portal is freely accessible and offering downloadable data sets, which can be accessed at cBioportal platform (https://www.cbioportal.org/study/summary?id=brca_tcga_pan_can_atlas_2018). The BRCA data set, publicly accessible through the TCGA database, was the primary data source utilized in this study.

## Supplementary Materials

The following supporting information can be downloaded at: https://www.mdpi.com/article/10.3390/ijms24065583/s1.
