# Supplementary material for: Chromatin Remodeling Enzyme Cluster Predicts Prognosis and Clinical Benefit of Therapeutic Strategy in Breast Cancer
Source: Int J Mol Sci. 2023 Mar 15;24(6):5583. doi: 10.3390/ijms24065583 (PMC10055970; doi:10.3390/ijms24065583)
Supplement: Supplementary file 1 [file ijms-24-05583-s001.zip › ijms-2207324-supplementary.pdf]

Supplemental Figures

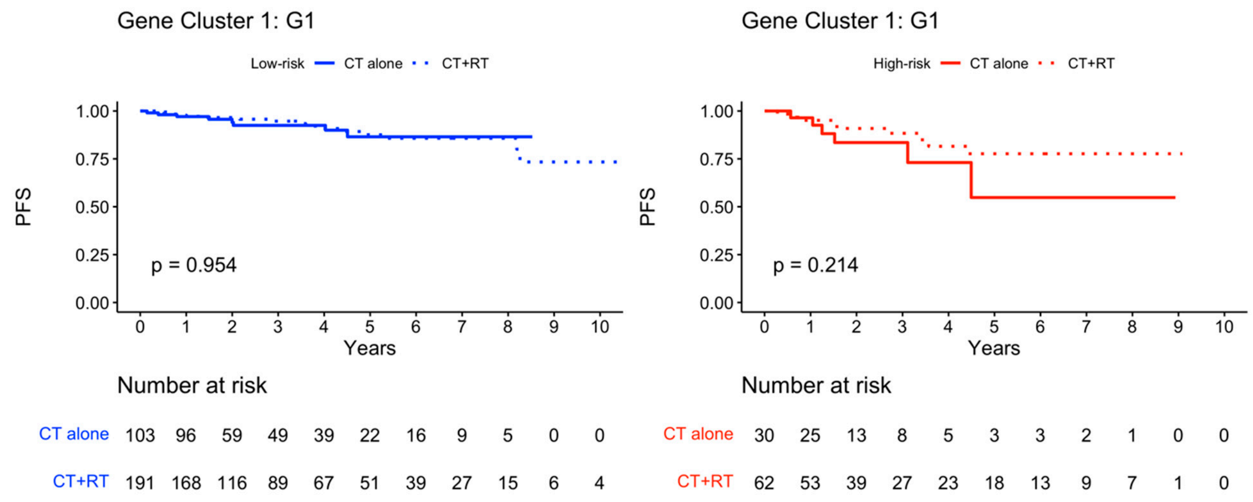

Figure S1. Kaplan–Meier plot for PFS according to treatment subgroup in different risk estimation subgroup of gene cluster 1. Blue solid line indicates CT alone and blue dotted line indicates CT with RT in low-risk group. Red solid line indicates CT alone and red dotted line indicates CT with RT in high-risk group. p-value is estimated using log-rank test.

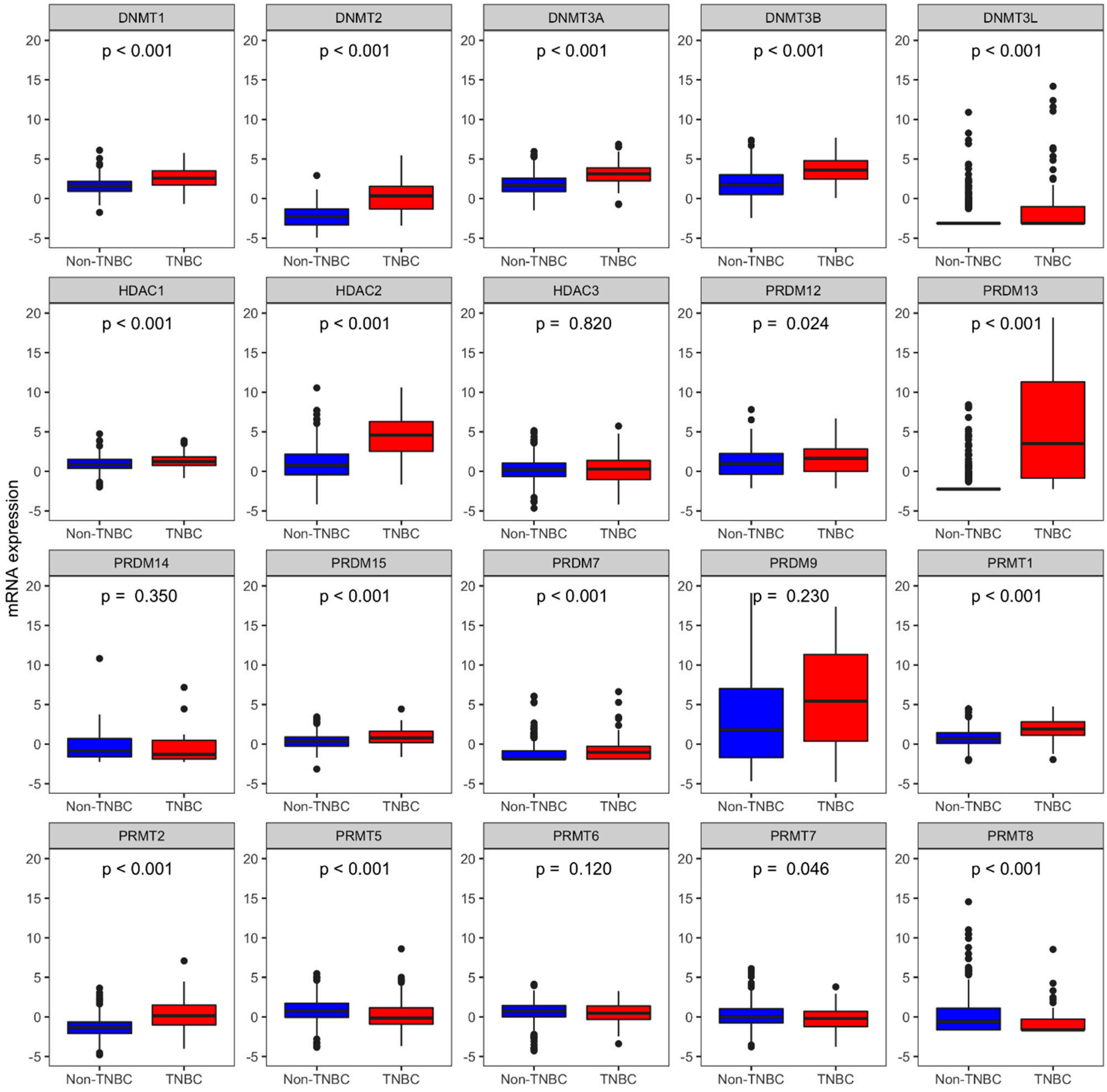

Figure S2. Boxplot of 20 target genes in gene cluster 1. Red color represents non-TNBC group, and blue color represents TNBC group. p-value is estimated using Wilcoxon rank sum test.

TNBC **non-TNBC**

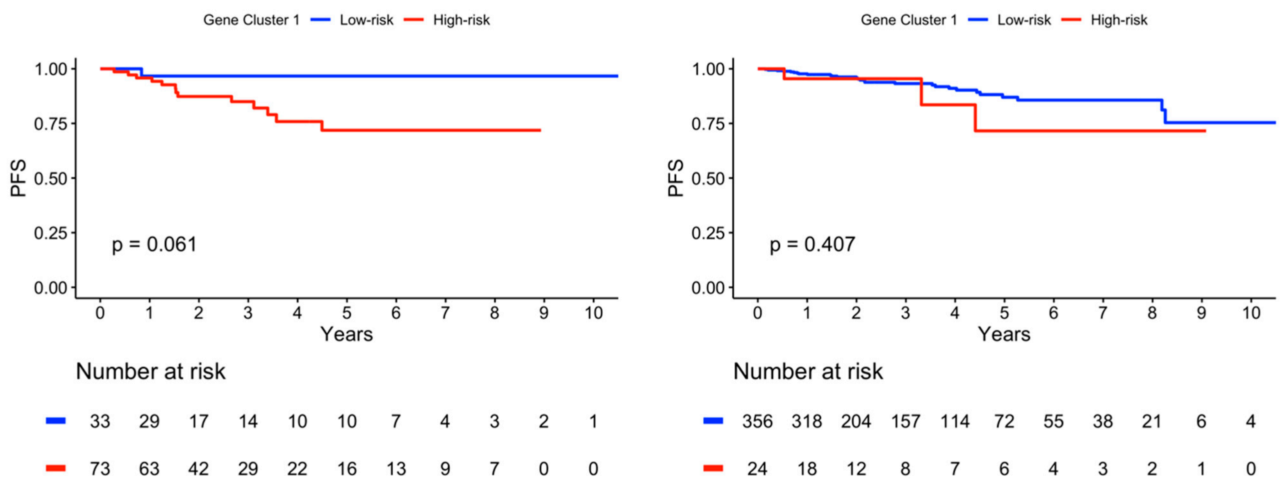

Figure S3. Kaplan-Meier plot for TNBC and non-TNBC PFS according to risk estimation subgroup of gene cluster 1. Red solid line indicates high-risk subgroup, and blue solid line indicates low-risk group. *p*-value is estimated using log-rank test.

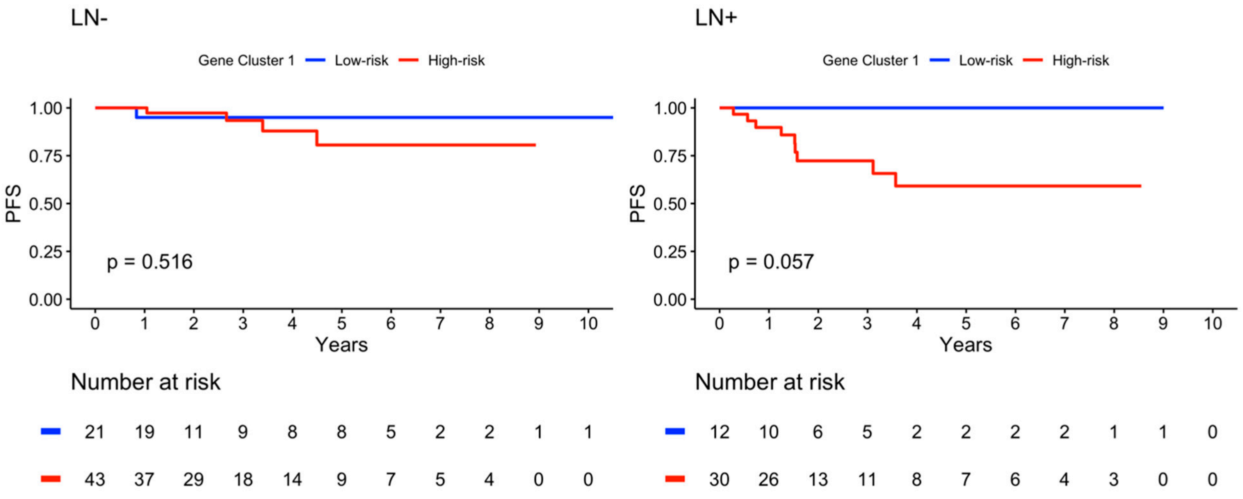

Figure S4. Kaplan-Meier plot for PFS according to risk estimation subgroup of gene cluster 1 in TNBC with different lymph node (LN) invasion status. Red solid line indicates high-risk subgroup, and blue solid line indicates low-risk group. *p*-value is estimated using log-rank test.

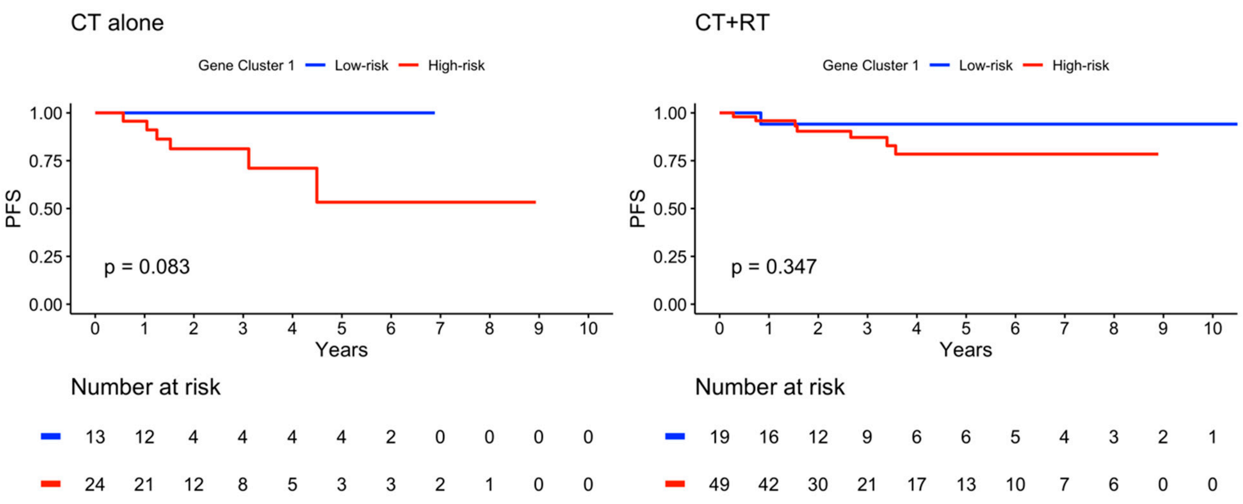

Figure S5. Kaplan-Meier plot for PFS according to risk estimation subgroup of gene cluster 1 in TNBC with different treatment subgroup. Red solid line indicates high-risk subgroup, and blue solid line indicates low-risk group. *p*-value is estimated using log-rank test.

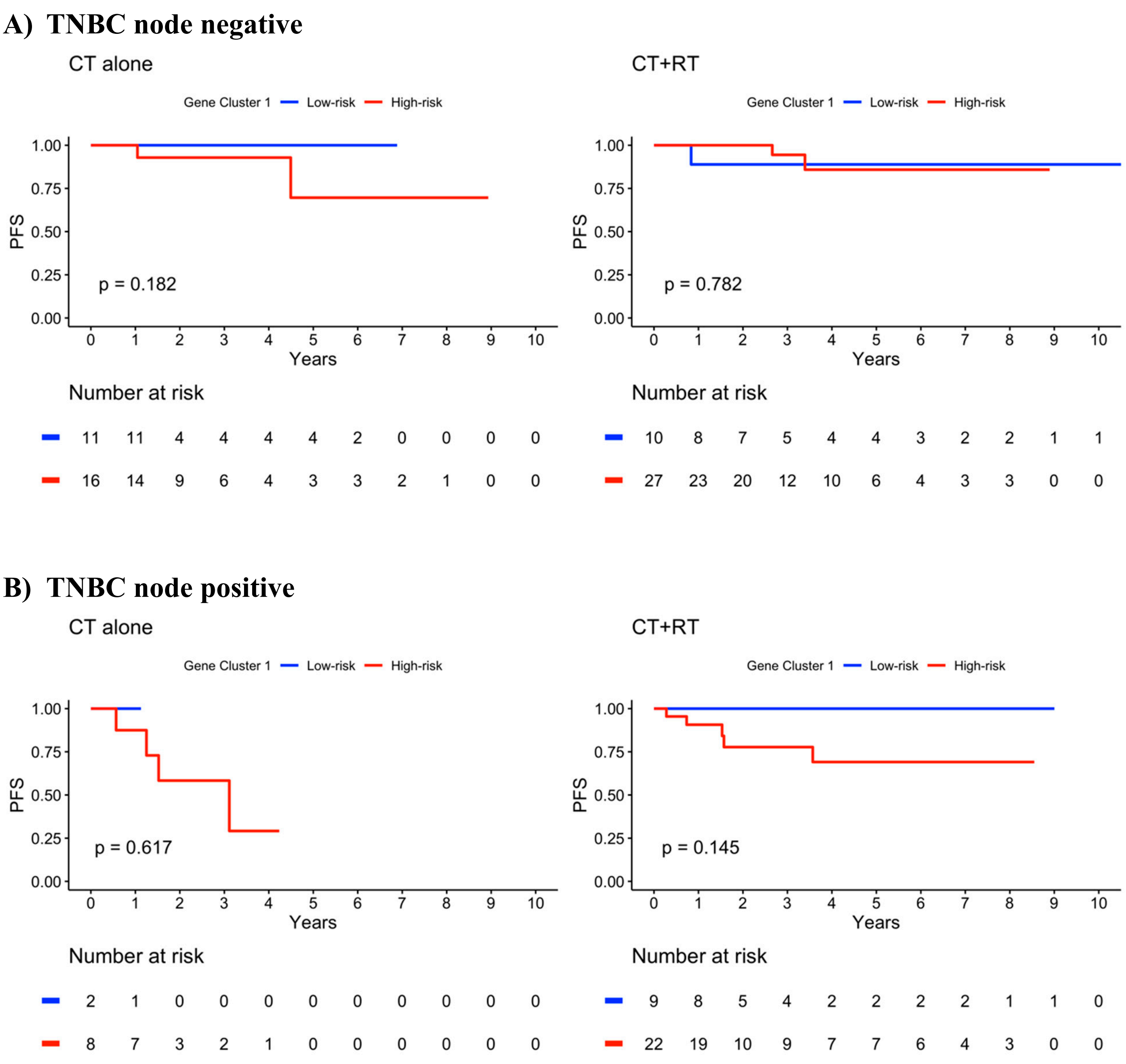

Figure S6. Kaplan-Meier plot for PFS according to risk estimation subgroup of gene cluster 1 in TNBC with A) TNBC node negative groups and B) TNBC node positive group in different treatment subgroup. Red solid line indicates high-risk subgroup, and blue solid line indicates low-risk group. *p*-value is estimated using log-rank test.

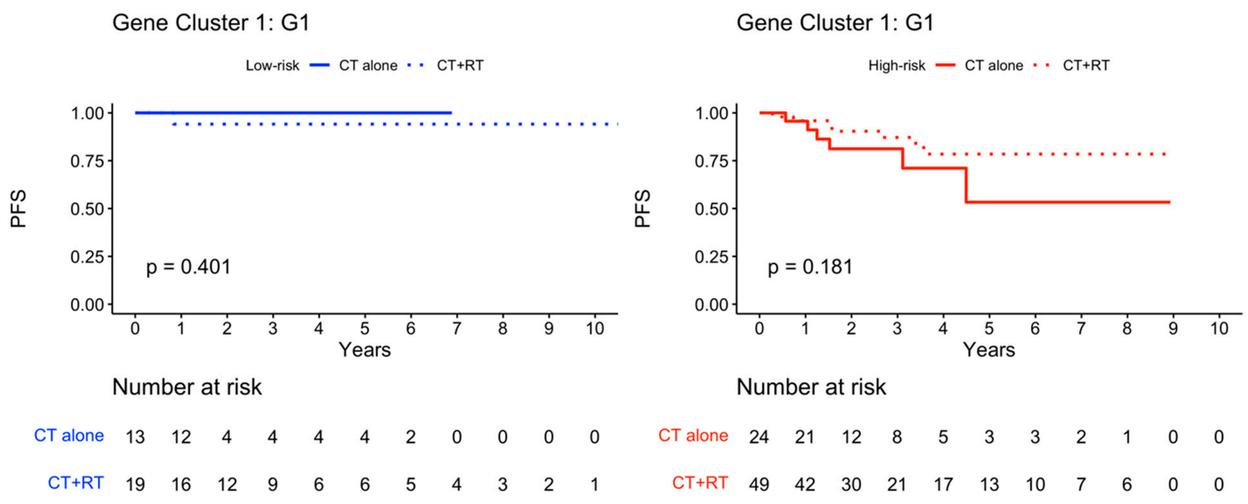

Figure S7. Kaplan-Meier plot for PFS according to treatment subgroup in different risk estimation subgroup of gene cluster 1. Blue solid line indicates CT alone and blue dotted line indicates CT with RT in low-risk group. Red solid line indicates CT alone and red dotted line indicates CT with RT in high-risk group. *p*-value is estimated using log-rank test.

A) TNBC node negative

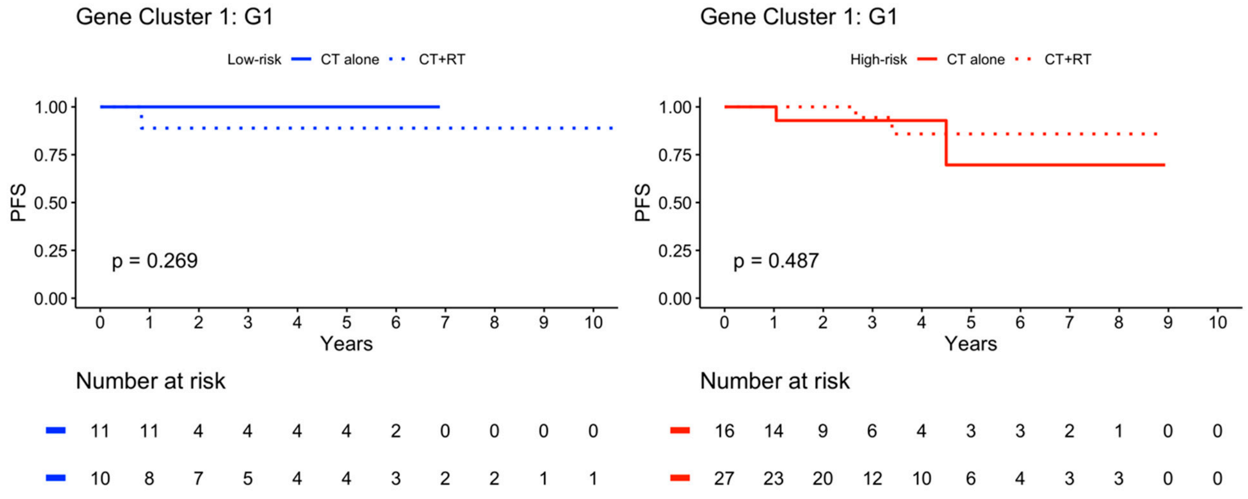

B) TNBC node positive

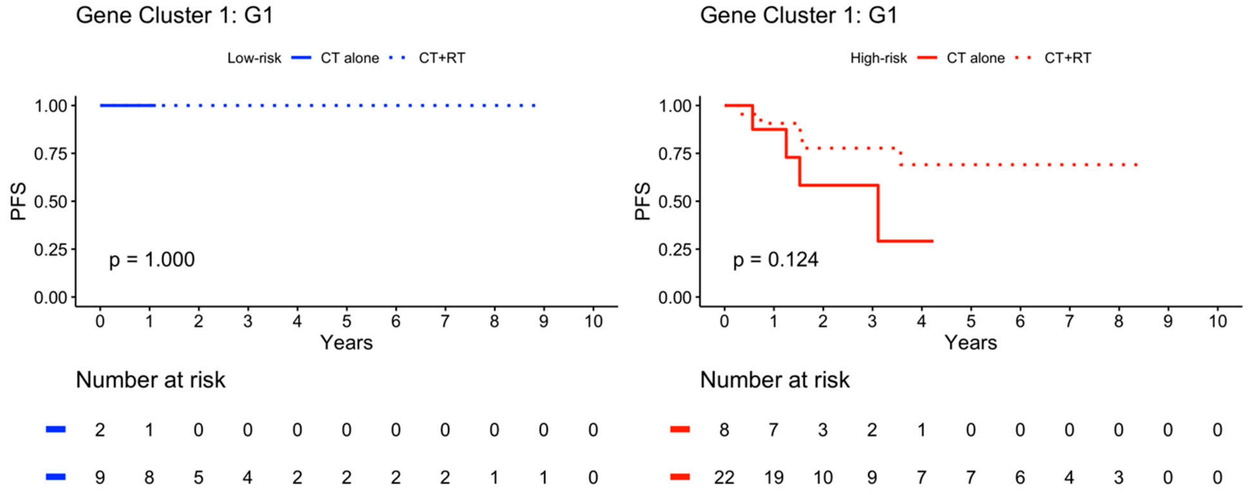

Figure S8. Kaplan-Meier plot for PFS according to treatment subgroup in different risk estimation subgroup of gene cluster 1 in TNBC with A) TNBC node negative groups and B) TNBC node positive group in different subgroup. Blue solid line indicates CT alone and blue dotted line indicates CT with RT in low-risk group. Red solid line indicates CT alone and red dotted line indicates CT with RT in high-risk group. *p*-value is estimated using log-rank test.

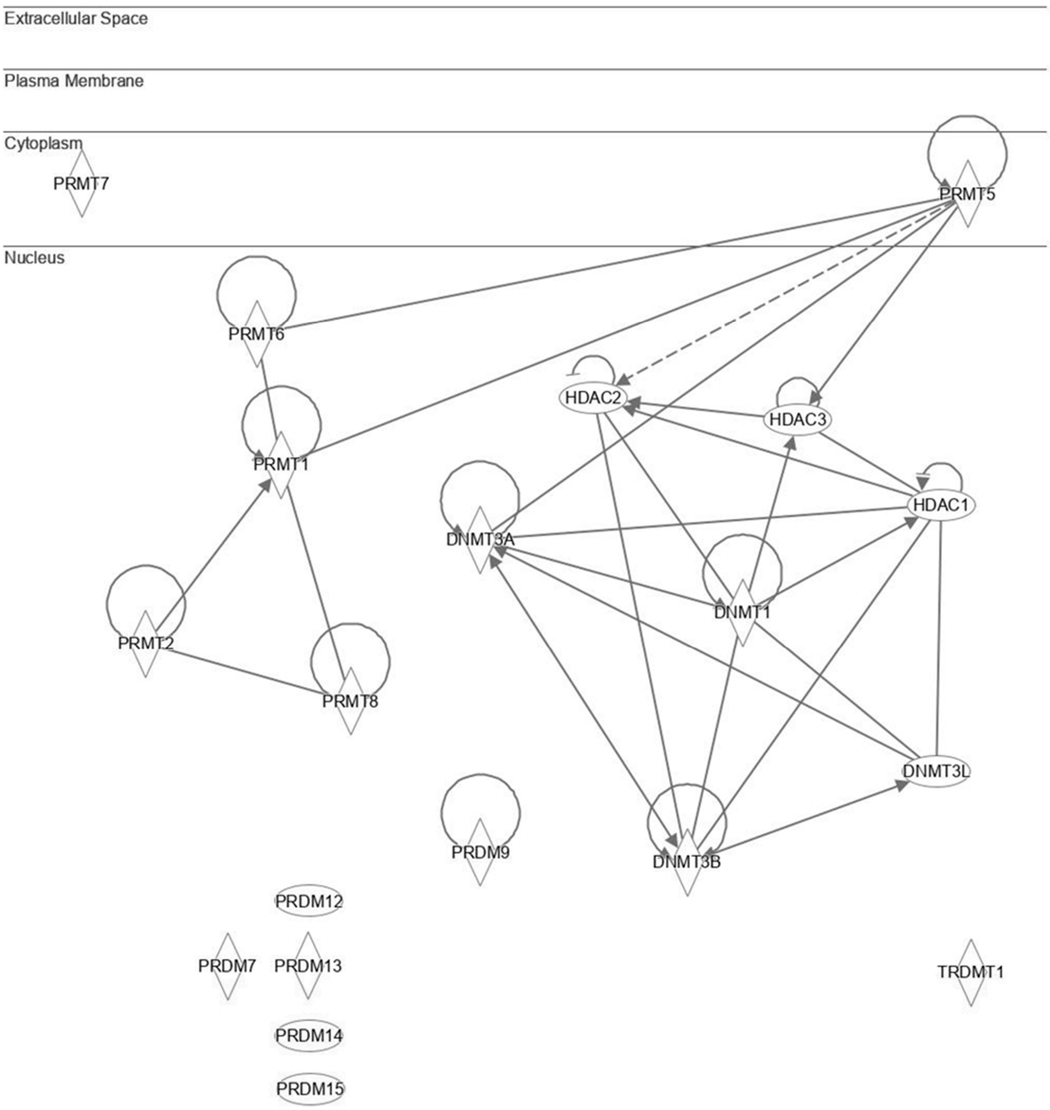

© 2000-2022 QIAGEN. All rights reserved.

Figure S9. Illustration of 4 gene family in our study

Table S1. mRNA expression of target genes according to risk estimation subgroup determined using gene cluster 2.

Genes	Gene cluster 2		*P*
	Low-risk (n = 279)	High-risk (n = 207)	
Gene cluster 2 included			
PRDM1	-0.23 (-4.65, 3.15)	1.20 (-2.45, 4.93)	**<0.001**
PRDM2	-2.20 (-8.84, 2.68)	-0.88 (-4.50, 2.85)	**<0.001**
PRDM4	0.29 (-7.29, 5.97)	0.70 (-6.08, 6.63)	**0.002**
PRDM5	-3.81 (-11.00, -0.26)	-2.14 (-8.38, 3.04)	**<0.001**
PRDM6	-0.77 (-4.58, 3.23)	0.63 (-2.52, 6.60)	**<0.001**
PRDM8	-3.35 (-9.36, 0.39)	-1.65 (-5.06, 3.47)	**<0.001**
PRDM10	-0.60 (-4.63, 3.37)	-0.18 (-2.09, 1.74)	**<0.001**
PRDM11	-1.65 (-5.57, 2.43)	-1.80 (-6.01, 1.69)	0.488
PRDM16	-3.49 (-6.56, 0.90)	-1.90 (-5.27, 2.62)	**<0.001**
PRMT3	1.14 (-5.64, 7.34)	0.41 (-4.00, 3.86)	**<0.001**
PRMT10	-1.77 (-8.33, 2.37)	-1.27 (-5.30, 2.63)	**<0.001**

All mRNA expression were summarized using median and range.

p-value is estimated using Wilcoxon rank-sum test.

Table S2. Distribution of baseline characteristics according to hierarchical cluster.

Characteristics	Gene cluster 1		*P*	Gene cluster 2		*P*
	Low-risk (n = 389)	High-risk (n = 97)		Low-risk (n = 279)	High-risk (n = 207)	
Diagnosis age (years), mean ± SD	56.7 ± 12.7	53.0 ± 10.6	0.003	56.8 ± 12.9	54.7 ± 11.7	0.059
Age ≥ 50yr	275 (70.7%)	54 (55.7%)	0.005	188 (67.4%)	141 (68.1%)	0.864
Age < 50yr	114 (29.3%)	43 (44.3%)		91 (32.6%)	66 (31.9%)	
Subtype			<0.001			<0.001
TNBC	33 (8.5%)	73 (75.3%)		79 (28.3%)	27 (13.0%)	
Luminal A	215 (55.3%)	9 (9.3%)		100 (35.8%)	124 (59.9%)	
Luminal B	108 (27.8%)	8 (8.2%)		90 (32.3%)	26 (12.6%)	
HER2 type	33 (8.5%)	7 (7.2%)		10 (3.6%)	30 (14.5%)	
Stage			0.766			0.210
Stage I	71 (18.3%)	16 (16.5%)		43 (15.4%)	44 (21.3%)	
Stage II	237 (60.9%)	63 (64.9%)		180 (64.5%)	120 (58.0%)	
Stage III	81 (20.8%)	18 (18.6%)		56 (20.1%)	43 (20.8%)	
IIIA	58 (14.9%)	12 (12.4%)		41 (14.7%)	29 (14.0%)	
IIIB	6 (1.5%)	0 (0.0%)		3 (1.1%)	3 (1.4%)	
IIIC	17 (4.4%)	6 (6.2%)		12 (4.3%)	11 (5.3%)	
Tumor size			0.208			0.009
T1 (<2cm)	121 (31.1%)	23 (23.7%)		69 (24.7%)	75 (36.2%)	
T2 (2cm-5cm)	234 (60.2%)	69 (71.1%)		183 (65.6%)	120 (58.0%)	
T3 (>5cm)	27 (6.9%)	5 (5.2%)		24 (8.6%)	8 (3.9%)	
T4 (direct to chest wall or skin)	7 (1.8%)	0 (0.0%)		3 (1.1%)	4 (1.9%)	
Lymph node status			0.065			0.602
Non-lymph node invasion (N0)	172 (44.2%)	53 (54.6%)		132 (47.3%)	93 (44.9%)	
Lymph node invasion (LN+)	217 (55.8%)	44 (45.4%)		147 (52.7%)	114 (55.1%)	
N1(1-3)	172 (44.2%)	53 (54.6%)		101 (36.2%)	77 (37.2%)	
N2(4-9)	151 (38.8%)	27 (27.8%)		33 (11.8%)	26 (12.6%)	
N3(≥ 10)	48 (12.3%)	11 (11.3%)		13 (4.7%)	11 (5.3%)	
Treatment						
Radiotherapy	242 (62.2%)	63 (64.9%)	0.618	178 (63.8%)	127 (61.4%)	0.581
Chemotherapy	294 (75.6%)	92 (94.8%)	<0.001	219 (78.5%)	167 (80.7%)	0.556
Treatment subgroup			0.003			0.469
CT alone	103 (26.5%)	30 (30.9%)		75 (26.9%)	58 (28.0%)	
RT alone	51 (13.1%)	1 (1.0%)		34 (12.2%)	18 (8.7%)	
CT + RT	235 (60.4%)	66 (68.0%)		170 (60.9%)	131 (63.3%)	
Survival outcome						
Died	19 (4.9%)	10 (10.3%)	0.044	20 (7.2%)	9 (4.3%)	0.194

Progressed	29 (7.5%)	16 (16.5%)	0.006	28 (10.0%)	17 (8.2%)	0.493

p-value is estimated using independent two-sampled t-test or chi-squared test.
